# Defective HIV-1 genomes and their potential impact on HIV pathogenesis

**DOI:** 10.1186/s12977-022-00601-8

**Published:** 2022-06-28

**Authors:** Jeffrey Kuniholm, Carolyn Coote, Andrew J. Henderson

**Affiliations:** 1 Department of Microbiology, Section of Infectious Diseases, Boston University School of Medicine, Boston, MA 02116 USA; 2 Department of Medicine, Section of Infectious Diseases, Boston University School of Medicine, Boston, MA 02116 USA

**Keywords:** Defective proviruses, Persistent reservoir, HIV latency, Transcription

## Abstract

Defective HIV-1 proviruses represent a population of viral genomes that are selected for by immune pressures, and clonally expanded to dominate the persistent HIV-1 proviral genome landscape. There are examples of RNA and protein expression from these compromised genomes which are generated by a variety of mechanisms. Despite the evidence that these proviruses are transcribed and translated, their role in HIV pathogenesis has not been fully explored. The potential for these genomes to participate in immune stimulation is particularly relevant considering the accumulation of cells harboring these defective proviruses over the course of antiretroviral therapy in people living with HIV. The expression of defective proviruses in different cells and tissues could drive innate sensing mechanisms and inflammation. They may also alter antiviral T cell responses and myeloid cell functions that directly contribute to HIV-1 associated chronic comorbidities. Understanding the impact of these defective proviruses needs to be considered as we advance cure strategies that focus on targeting the diverse population of HIV-1 proviral genomes.

**Graphical abstract:**

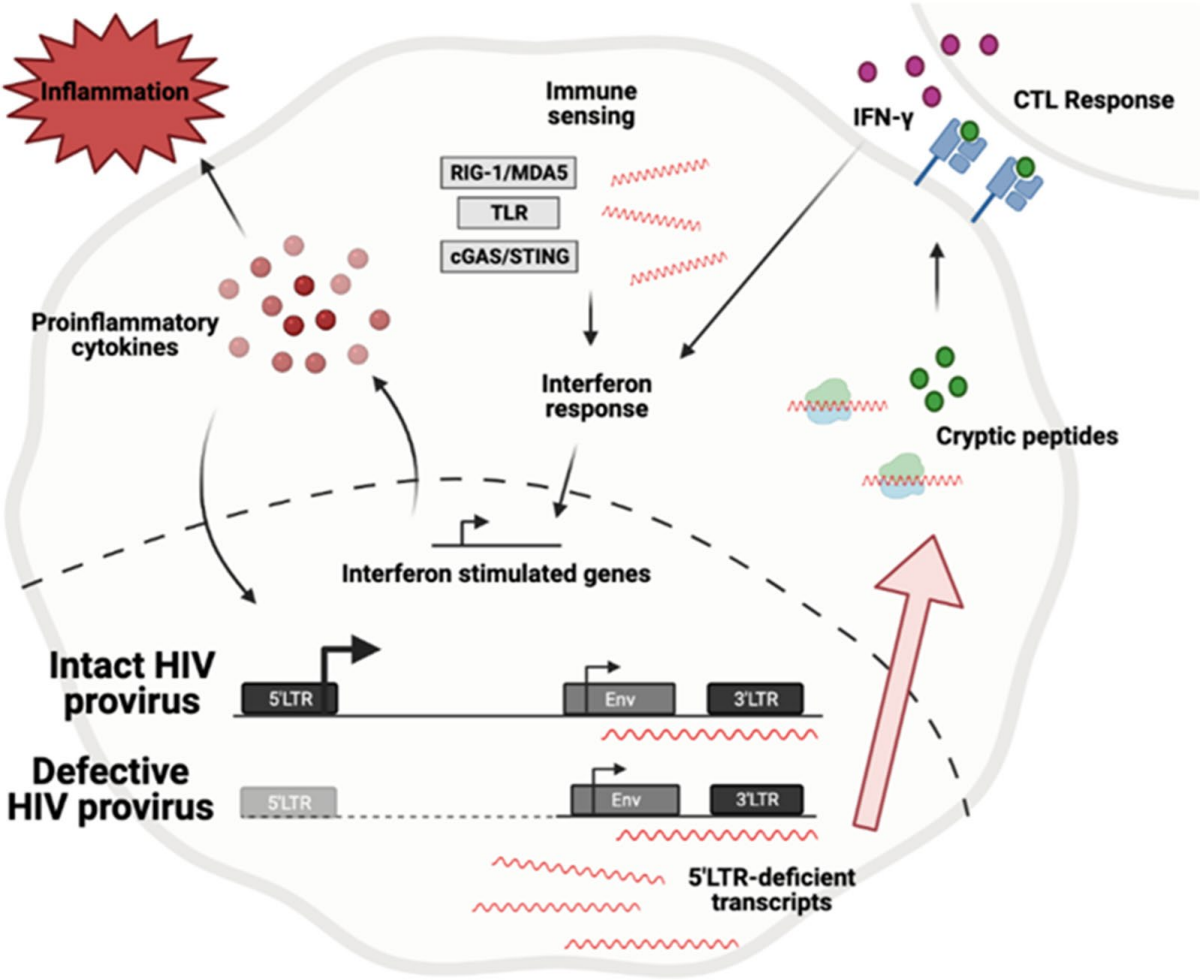

## Introduction

Virus replication requires successful entry into a host cell, generation of the viral genome, packaging of virion contents, and transmission of these contents to a new target cell. However, intrinsic host cell restriction factors and the inefficient and error-prone nature of viral replicative processes lead to the generation of defective virus genomes and particles. Defective viruses are generated by several RNA viruses including Measles, Sendai and Ebola Viruses [1–7]. They are also generated by retroviruses including human immunodeficiency virus-1 (HIV-1) [8–10], the focus of this review. Defective proviruses accumulate crippling mutations during infection and replication which render them unable to complete their replication cycle. Despite their inability to contribute to new infections, these defective viruses still potentially influence viral pathogenesis by diverting productive anti-viral immunity and propagating damaging inflammatory responses. Therefore, defective viruses may be critical contributors to viral immune escape, persistence, and pathogenesis and not simply viral genome “junk”.

The focus of HIV cure strategies has primarily been on eliminating or suppressing the intact latent provirus genomes that fuel the rebound of HIV replication upon interruption of antiretroviral therapy [11, 12]. However, the number of intact proviral genomes that are small, estimated to be 2% of all infected cells which includes a rarer population that contributes to viral rebound (one in 100,000 to 1 × 10^6^ cells in peripheral blood and lymph nodes) in people living with HIV (PLWH) on antiretroviral therapy (ART). Secondary lymphoid tissues also harbor HIV-1 infected CD4+ T cells with frequencies of intact, defective and inducible proviral genomes similar to those observed in blood suggesting peripheral blood is an appropriate surrogate for evaluating persistent proviral sequences [13]. Furthermore, intact and defective proviral genomes are found in most CD4+ T cells subsets in comparable frequencies indicating multiple CD4+ cell types in multiple tissues contribute to HIV-1 persistence and latency [14]. However, it will be critical to have standardized clinically validated assays to evaluate latent reservoirs and persistent proviral genomes in blood and immune tissues to monitor the effectiveness of therapeutic approaches for HIV-1 cure [15].

With the majority of proviral sequences harboring deleterious mutations [8] how these defective HIV proviruses contribute to various persistent comorbidities and pathogenesis in people living with HIV remains an important unanswered question. When considering different cure strategies, whether genome editing to cripple HIV-1, “block-and-lock”, or “shock-and-kill” approaches, it will be critical to determine whether remnants of viral genomes are expressed and biologically active. In this review we highlight findings that demonstrate that HIV infection results in dynamic populations of defective genomes, discuss the expression and evolution of these defective proviruses in PLWH, and consider whether their expression contributes to HIV immune evasion, persistent inflammation, and pathogenesis.

## Defective HIV-1 genomes dominate the proviral landscape

Characterization of the HIV-1 proviral reservoir in different CD4+ T cell subsets through deep sequencing, single-cell approaches, ex vivo viral outgrowth assays, and quantitative droplet digital PCR has led to insights into how persistent infection and latency are established, maintained, and reactivated [8, 9, 14, 16–23]. From these efforts it has become apparent that the proviral landscape is dynamic and evolving during chronic HIV-1 infection [24–29]. Intact HIV-1 proviruses which have the potential to support viral rebound, have been estimated to represent 2–5% of the persistent provirus pool as measured in peripheral blood mononuclear cells (PBMCs) [8, 9]. Longitudinal tracking of intact provirus sequences in PLWH before and after ART initiation suggests that most of the latent intact HIV reservoir has been seeded at the time of ART initiation and there is no additional infection post-ART [30–32], although ART initiation has been suggested to shape the latent reservoir [30–32]. It has also been suggested that the virus circulating at the time of ART initiation is overrepresented in the reservoir [30]. The size of the persistent provirus population varies among individuals and the mechanisms that determine if a proviral genome is capable of reactivation remain inadequately understood. Studies of CD4+ T cells from individuals who naturally control HIV-1 infection demonstrated that intact proviruses are enriched in heterochromatic regions of the host genome while defective proviruses are detected in euchromatic regions [19, 33]. These observations support that enhanced immune detection and clearance in these individuals shapes the persistent provirus reservoir over time, relegating intact proviruses to relatively silent loci of the host genome [34]. Examining the reactivation of provirus from peripheral blood obtained from PLWH that are undergoing ART indicates that relatively small subsets of latently infected cells are easily induced to express new virions while a second larger subset of infected cells harbor intact provirus that are more resistant to reactivation [9]. The mechanisms that are responsible for this spectrum of inducibility of intact proviruses is unclear and may reflect phenotypes and functions of cells that harbor HIV-1 infections, proviral integration sites, or even stochastic mechanisms such as bursts of Tat-dependent transcriptional activity [35–42].

The majority of proviral sequences detected, greater than 90%, are defective [8, 9, 16]. These defective genomes harbor large deletions, sequence inversions, hypermutations, and defective splice donor and acceptor sites that prevent viral replication. During the course of treatment, the persistent proviral landscape shifts with outgrowths of dominant clones that include defective proviral genomes [24]. Proposed mechanisms that drive the shaping, selection, and expansion of HIV-1 proviral clones include depletion of cells that express HIV antigens, antigen driven and cytokine driven clonal expansion, homeostasis of T cell subsets that harbor HIV proviruses, and expansion of proviruses integrated near genes that influence cell survival and proliferation [24, 25, 43–47]. Longitudinal studies have revealed that defective proviruses are subjected to different levels of immunological targeting and clearance depending on their transcriptional and translation competence [24, 26, 48]. Proviruses which retain the ability to transcribe HIV-1 RNAs and translate viral proteins can be preferentially cleared during sustained immunological pressure [29] leading to proviruses with little transcriptional or translational activity clonally expand to form the majority of the reservoir [24, 25, 29]. However, HIV-1 proviral genomes that are transcriptionally active and express gag have also been posited to drive clonal expansion [25]. Sequencing proviral genomes have suggested that persistent defective proviruses are established within the first few weeks following infection, although initiation of antiretroviral therapy may influence repertoire of defective HIV proviral sequences [8, 26]. It remains unclear whether defective proviruses play a role in subverting the anti-HIV immune responses or perpetuating the chronic inflammation which has been described in PLWH on ART.

## Generation of defective HIV-1 genomes

Multiple mechanisms contribute to the generation of defective HIV-1 proviruses including the inefficiency of reverse transcription and the activity of host cell restriction factors. HIV reverse transcriptase lacks proofreading ability and is error prone, introducing approximately 1.4 × 10^–5^ mutations per base pair per cycle [49, 50]. Successful reverse transcription also requires dissociation and re-initiation of reverse transcription on the RNA genome template leading to a propensity to produce mutated and truncated HIV DNA intermediates [51–53]. For example, sequence analysis of HIV proviral sequences obtained from CD4+ T cells from PLWH on ART attributed approximately 40% of the internal deletions detected to negative strand synthesis during reverse transcription [54]. Recombination, a process by which genetic diversity is introduced through template-switching between the two copies of the HIV RNA genome packaged in virions, also contributes to the mutation rate of reverse transcription products [55–58].

Intrinsic host defenses and anti-viral restriction factors limit replication and reverse transcription efficiency contributing to the generation of defective HIV genomes. APOBEC 3G, a cytosine deaminase, targets single-stranded DNA intermediates and promotes HIV-1 hypermutation by inducing guanine-to-adenine changes during the process of reverse transcription [59–63]. Sterile Alpha Motif- and HD-domain containing protein 1 (SAMHD1), a host viral restriction factor which reduces the concentration of intracellular nucleotides in resting CD4+ T cells and myeloid cells, limiting the efficiency and completion of reverse transcription [64–67].

## RNAs and translation products from intact and defective HIV-1 proviruses

HIV-1 transcription is regulated by multiple mechanisms and combinatorial events which have been extensively reviewed (recent reviews include [68–70]). In general, the HIV-1 long terminal repeat (LTR) acts as an enhancer and promoter, recruiting host cell transcriptional activators, repressors, chromatin remodeling factors, and the RNAP II complex which all influence transcriptional activation or repression. HIV Tat binds the TAR stem loop element at the 5ʹ end of the HIV-1 initiated transcript to recruit PTEFb a cofactor that enhances RNAPII processivity and recruits cofactors that influence proviral chromatin organization and transcription [71–73]. Current antiretroviral therapies do not target HIV transcription; however, during ART there is immune selection against cells actively transcribing HIV genes. The function of intrinsic transcription factors and repressive epigenetic regulators contribute to the repression of HIV-1 transcription in intact latent proviruses. However, it is important to note that HIV-1 transcripts are detected in individuals on ART [74–77]. Single-genome HIV RNA sequencing at limiting dilution showed that up to 7% of HIV-1 provirus in PBMCs from patients undergoing ART remain transcriptionally active [78].

**Fig. 1:**
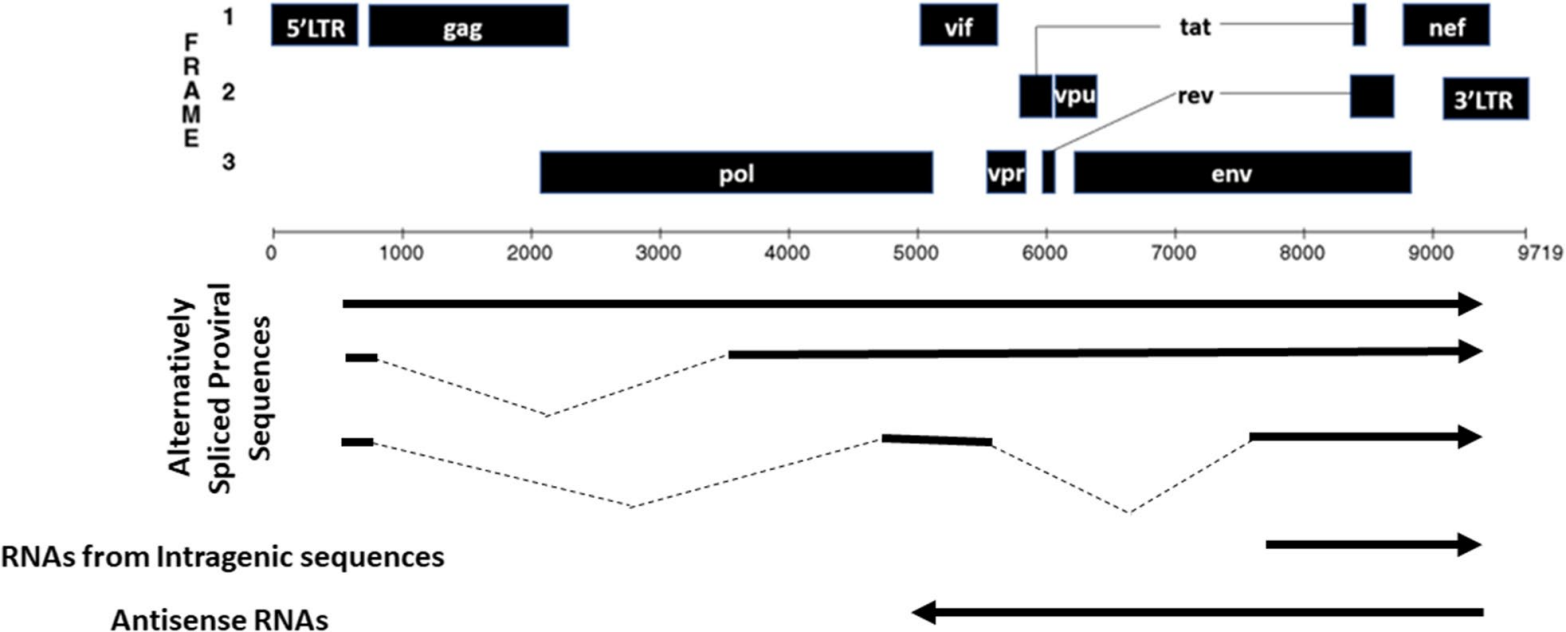
Summary of a subset of RNAs that are transcribed by HIV-1 outlined in the review. Dashes represent spliced sequences

A potential source of residual HIV-1 transcripts detected during ART are defective proviruses (Fig. 1). Defective HIV-1 proviruses are transcribed despite mutations that compromise efficient transcription and replication such as deletions of the 5ʹLTR or altered splice acceptor and donor sites, including the psi packaging element [24, 54]. Despite these defects, spurious transcription, possibly through alternative transcriptional start sites and/or alternative splice site usage, has been reported [24]. Proviruses with defects in their major splice donor sequence overcome this defect by using alternative splicing mechanisms. The use of alternative and cryptic splice sites is suspected to enable translation of chimeric and non-canonical HIV-1 fusion proteins [79]. Antisense transcription from the 3ʹ LTR is another mechanism for generating HIV-1 transcripts; however, whether antisense transcription is regulated by the same signaling cascades as transcription from the 5ʹ LTR, and its functional relevance, is unclear [80–83].

Intragenic cis-acting elements have been proposed for HIV-1 and other retroviruses and represent additional mechanisms to support the transcription of defective proviruses [84–88]. The presence of intragenic transcriptional elements in the HIV-1 genome has been postulated for decades but the function and regulation of such elements have not been fully appreciated. Cis-acting repressive sequences (CRS) have been reported and have been proposed to limit HIV-1 transcription, splicing, and nuclear export [89, 90]. CRS functions are partially achieved through interactions with host cellular transcription factors [90]. Such interactions have also been described for cis-acting elements involved in regulating the alternative splicing of HIV-1 transcripts [91]. In addition, sequences within the HIV-1 env gene have been identified as potential elements that control intragenic transcriptional activity and include transcription binding sites, the presence of methylated CpG islands, and increased DNAse I sensitivity which correlates with transcriptionally active elements [92–94]. We have extended these observations using 5ʹ RACE PCR to demonstrate that HIV transcripts are generated from an intragenic promoter within the envelope gene in in vitro infected primary cells [95]. Potential aberrant RNAs that contained env and nef but lacked 5ʹ LTR derived untranslated regions (UTRs) were detected in cDNAs generated from cell-associated RNA from PLWH on ART using multiplex reverse transcriptase droplet digital PCR [92]. A limitation with cDNA synthesis is that prematurely terminated cDNAs molecules would be included in the library. However, taken together these results lead to speculation that spurious transcription driven by cis-acting elements that remain active in defective HIV-1 proviruses could provide a mechanism for the generation of RNA when LTR-mediated transcription is repressed or compromised. Whether this transcription from defective proviruses is relevant to HIV-1 pathogenesis is an outstanding question as are mechanisms that regulate these intragenic promoters.

A critical question regarding potential roles of these cryptic or alternative RNA sequences is whether they are translated. It has been shown that point mutations within the HIV-1 provirus generate alternative reading frames and these can allow for translation of proteins [96, 97]. Similarly, internal deletions and inversions within defective proviral genomes can generate novel open reading frames and translation of proteins [54, 98]. HIV-1 proteins are also translated from transcripts generated from intragenic promoters and there have been reports of an antisense protein [80, 82, 95, 99]. Translation from these aberrant or spurious RNAs would be consistent with the detection of HIV-1 proteins in PWLH on ART and in latently infected cells in the absence of viral replication. For example, Nef and Gag have been observed intracellularly in PBMCs from PLWH on ART, although technical concerns have been raised about these studies and protein detection has often required ex vivo stimulation [100–102]. HIV-1 Gag has been shown to be a source of defective ribosomal products (DRiPs) which are rapidly degraded by the proteasome and loaded onto MHC-I molecules [103]. HIV-1 antisense protein (ASP) has been reported in infected cell samples from PLWH and antibodies against this protein have been detected in the sera of a subset of infected individuals [82, 104, 105]. The possible generation of viral transcripts and proteins from defective HIV-1 proviruses begs the question of whether these viral products play an immunomodulatory role in chronically infected individuals.

## Defective viruses, immune dysfunction, and cure strategies

**Fig. 2:**
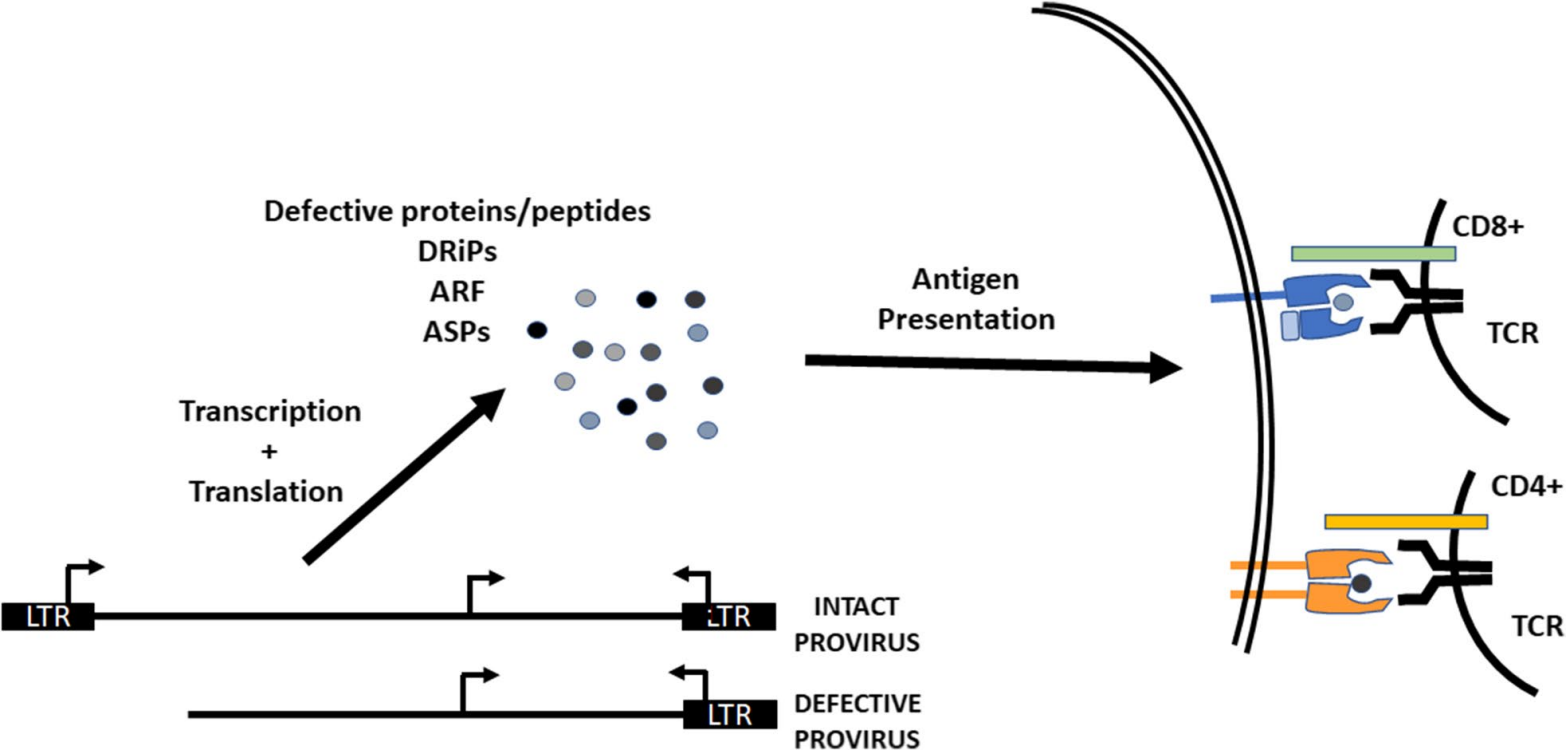
Mechanisms by which cryptic HIV-1 peptides could influence T cell responses

Immunological selection and clonal expansion of cells harboring HIV proviral genomes shapes the persistent reservoir [24, 33, 46, 106, 107]. For example, cells that express HIV-1 generate MHC-I associated peptides that are targeted and eliminated by CD8+ T cells. This pool of HIV peptides includes cryptic epitopes produced from alternative reading frames (ARFs) throughout the HIV-1 genome [97, 105, 108, 109]. ELISA and ELISpot assays demonstrated that CD8+ T cells from PLWH on ART are activated by peptides predicted to be generated by ARFs which exist in sense and antisense orientations in the HIV-1 genome [24, 105]. These responses were greater in magnitude for CD8+ T cells from chronically infected PLWH as opposed to those with an acute infection. These data support a model whereby ARFs shape the composition of persistent proviruses by being targets for CD8+ T cells and driving the homeostasis of CD8+ T cell-mediated immunity against these cryptic proteins. Studies that have demonstrated the ability of defective proviruses to produce viral proteins suggest that subsets of the defective provirus population could contribute to this phenomenon [98, 101, 102]. We speculate that defective proviral genomes act as one source of ARF generated peptides. Ex vivo expression of defective HIV-1 provirus clones has shown that defective HIV-1 genomes can be translated into proteins which activate cytotoxic T lymphocytes (CTLs) specific for HIV-1 peptides [24]. These data support that the adaptive immune response is influenced by protein expression from a subset of defective proviruses (Fig. 2).

One consequence of chronic HIV infection is the functional exhaustion of HIV-specific CD8+ T cells [110, 111]. Polyfunctional HIV-specific CD8+ T cells, those which produce a wide breadth of cytokines, chemokines, and cytotoxic molecules, correlate with control of HIV-1 in non-progressors who control HIV infection [110, 111]. Longitudinal analysis of polyfunctional HIV-specific CD8+ T cells in vivo has shown that, in the context of persistent antigen stimulation, the breadth of cytokines produced declines, and this correlates with increased expression of PD-1, TIGIT, and LAG-3, molecules associated with exhaustion [110, 112, 113]. While HIV-1 specific CD8+ T cell numbers remain high in chronically infected individuals, they produce less IFNγ, express relatively high levels of co-inhibitory receptors like PD-1, and have altered metabolomic profiles [114–116]. Additionally, CD4+ T cell depletion promotes exhaustion by diminishing helper T-cells that facilitate antiviral CD8+ T cell responses [117, 118]. In addition, there is evidence that cryptic peptides may have the capacity to drive viral escape from cellular immunity driving escape mutations which prevent proteasomal cleavage and antigen presentation of these otherwise protective epitopes [119]. Therefore, during chronic HIV infection, spurious and chronic antigen expression from the defective persistent provirus pool could subvert anti-HIV immunity by driving T cell exhaustion, diversion and depletion.

Chronic HIV infection is associated with persistent inflammation which has been implicated in various comorbidities and associated HIV-1 disease sequela consistent with inflammaging in PLWH [120]. For example, PLWH receiving ART have increased risk of coronary heart disease, various cancers, HIV-associated neurological disorders (HAND), leaky gut syndrome, and other end-organ diseases [121–127]. These HIV associated conditions have been correlated with an accumulation of age-related epigenetic marks in cells from the blood and brain leading to the hypothesis that HIV-1 infection promotes accelerated aging [128–131]. Importantly, the inflammaging phenomenon does not correlate with plasma viremia and is observed in PLWH even when viremia is largely controlled by ART. This inflammatory response may be driven by recognition of HIV proteins and RNAs activating innate intracellular antiviral responses. Markers of chronic inflammation in PLWH on ART do not correlate with measurements of intact provirus genomes but do correlate with cell associated HIV-1 RNA [132]. Whether residual transcription from defective proviral genomes contribute to this inflammation is undefined.

**Fig. 3:**
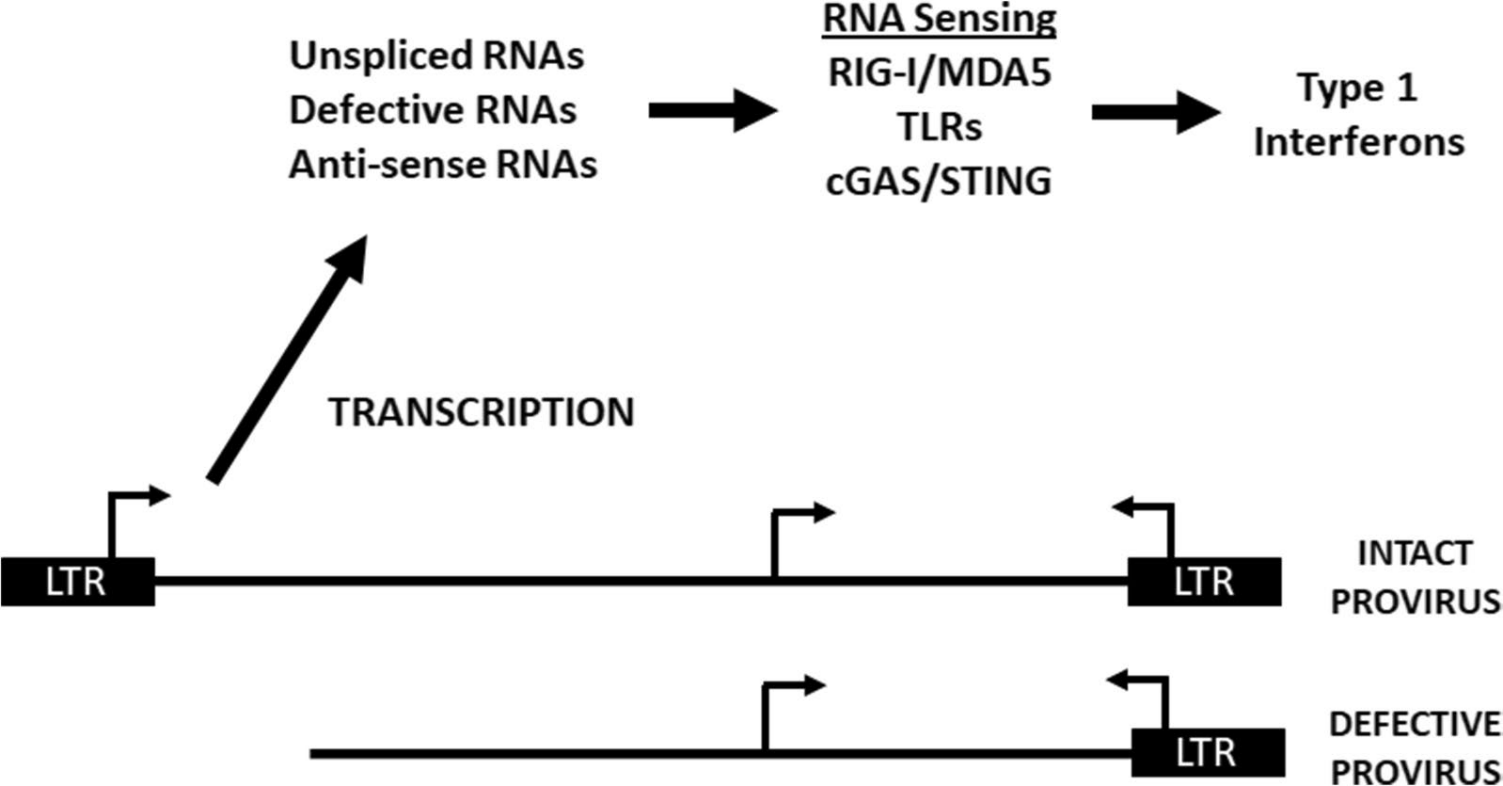
Mechanisms by which HIV-1 RNAs potentially activate innate immune responses

HIV-1 proviruses generate a diverse set of transcripts which include RNAs with complex secondary structures, retention of intronic sequence, and post-transcriptional modifications including m6A modifications [133–136]. These features of HIV-1 transcripts provide multiple targets for detection by cellular innate nucleic acid innate immune sensors which can initiate signaling events that activate interferon responses and inflammatory cytokine production [133, 135, 137–141]. For example, the expression of intron-containing HIV-1 RNA exported from the nucleus in infected myeloid cells and microglia has been demonstrated to perpetuate inflammatory responses [134, 135]. Detection of these RNAs and the induction of IFN type 1 responses alter macrophage and dendritic cell function (Fig. 3) including antigen presentation thus influencing CD4+ and CD8+ T cell responses. Together, these studies support that the residual transcription described in PLWH on ART have the potential to contribute to CD8+ T cell dysfunction and systemic inflammation.

Current cure strategies focus on either purging the HIV-1 provirus reservoir, permanently inactivating latent proviruses, or targeting the provirus with gene editing approaches [12, 142–147]. Examples of some these proposed approaches include shock-and-kill to activate the latent pool so it can be immunologically targeted, block-and-lock approaches that rely on compounds or engineered transcriptional repressors that inactivate or repress HIV proviral transcription such as didehydro-Cortistatin, dCas9-KRAB or dCasDMNTs and targeting and inactivating proviruses using CRISPR-cas9 or zinc finger nucleases [148–158]. These approaches, in general, target the expression or elimination of intact proviruses and would have minimum impact on the presence of defective proviruses. Since there is scant information as to how these defective proviral sequences are transcriptionally regulated, it is unknown whether latency reversal agents or transcriptional repressors will impact the activity of intragenic cis-transcriptional elements and the expression of cryptic peptides. Depending on the sequences targeted by engineered nucleases, gene editing approaches have the potential to create additional defective proviral genomes. Furthermore, CRISPR–cas9 approaches have been reported to promote viral escape through nonhomologous end joining and generate transcriptionally active LTR circles [159, 160]. As we explore ways to target the latent reservoir, continued understanding of the regulation and functional impact of defective proviruses need to be considered.

## Conclusions

The persistent HIV-1 proviral genome landscape consists of mostly defective HIV-1 proviruses. Although RNAs and proteins are expressed from these proviral genomes their impact in HIV pathogenesis is unclear. We speculate that spurious expression of these RNAs and proteins contribute to immune dysfunction and T cell exhaustion that are associated with comorbidities of chronic HIV-1 infection including inflammaging. Future cure strategies will need to address the importance of targeting the complete array of intact and defective proviral genomes.

## Data Availability

Not applicable.
